# Elevated body swing test after focal cerebral ischemia in rodents: methodological considerations

**DOI:** 10.1186/s12868-015-0189-8

**Published:** 2015-08-05

**Authors:** Edvin Ingberg, Johanna Gudjonsdottir, Elvar Theodorsson, Annette Theodorsson, Jakob O Ström

**Affiliations:** Division of Microbiology and Molecular Medicine, Department of Clinical and Experimental Medicine, Department of Clinical Chemistry, Faculty of Health Sciences, Center for Diagnostics, Linköping University, Region Östergötland, Linköping, Sweden; Division of Neuroscience, Department of Clinical and Experimental Medicine, Faculty of Health Sciences, Department of Neurosurgery, Anaesthetics, Operations and Specialty Surgery Center, Linköping University, Region Östergötland, Linköping, Sweden; Vårdvetenskapligt Forskningscentrum/Centre for Health Sciences, Örebro University Hospital, County Council of Örebro, Örebro, Sweden; School of Health and Medical Sciences, Örebro University, Örebro, Sweden

**Keywords:** Brain infarction, Focal cerebral ischemia, Middle cerebral artery occlusion, Elevated body swing test, Rodents, Rats, Lateralization

## Abstract

**Background:**

The elevated body swing test (EBST) is a behavioral test used to evaluate experimental stroke in rodents. The basic idea is that when the animal is suspended vertically by the tail, it will swing its head laterally to the left or right depending on lesion side. In a previous study from our lab using the EBST after middle cerebral artery occlusion (MCAo), rats swung contralateral to the infarct day 1 post-MCAo, but ipsilateral day 3 post-MCAo. This shift was unexpected and prompted us to perform the present study. First, the literature was systematically reviewed to elucidate whether a similar shift had been noticed before, and if consensus existed regarding swing direction. Secondly, an experiment was conducted to systematically investigate the suggested behavior. Eighty-three adult male and female Sprague–Dawley rats were subjected to MCAo or sham surgery and the EBST was performed up to 7 days after the lesion.

**Results:**

Both experimentally and through systematic literature review, the present study shows that the direction of biased swing activity in the EBST for rodents after cerebral ischemia can differ and even shift over time in some situations. The EBST curve for females was significantly different from that of males after the same occlusion time (p = 0.023).

**Conclusions:**

This study highlights the importance of adequate reporting of behavioral tests for lateralization and it is concluded that the EBST cannot be recommended as a test for motor asymmetry after MCAo in rats.

## Background

Stroke is one of the most devastating diseases in terms of mortality and disability [1]. In addition to the effects on patients and relatives, it imposes a substantial economic burden on the society [1]. Despite major research efforts providing improved understanding of stroke mechanisms the available treatment options are still few [2]. Preclinical stroke studies are mainly performed in rodents subjected to ischemic stroke, most commonly by middle cerebral artery occlusion (MCAo). The outcome measure, for example to evaluate the effect of a drug, has traditionally been infarct size but it is nowadays common to also include behavioral testing as an endpoint. Numerous such tests have been developed with the intention of examining how the stroke-induced animal is affected regarding sensory, motor or cognitive function or the emotional status [3].

Among the tests used for evaluation of motor functions is the elevated body swing test (EBST). Although a similar procedure was described as early as 1979 [4], the test in its current form was developed 1995 by Borlongan and Sanberg as a test for asymmetrical motor behavior in an animal model of Parkinson’s disease [5]. Soon after this, the EBST was employed by the same research group also in animal studies of Huntington’s disease [6] and ischemic stroke [7]. The basic idea is that when the animal is lifted by the tail and held vertically, it will swing its head to the left or to the right. Whereas healthy animals on a group level are likely to swing approximately 50% to either side, animals with a unilateral cerebral lesion, e.g. ischemic stroke, should present with a dominant/biased swing direction. The extent of such biased swing activity, for example overweight of swings to the rats’ right side, is suggested to be a measure of lateralization of motor behavior. The first stroke study using EBST described swings favoring the side ipsilateral to the lesion, and the test has subsequently been used to evaluate lesions in a number of preclinical ischemic stroke studies with rats [7–32], mice [33–35] and gerbils [36–38], as well as to confirm successful surgery for inclusion [39] (results from all these studies are presented herein). The two widely used neurological scoring exams for MCAo studies designed by Bederson [40] and Garcia [41], are also somewhat related to the EBST; both of these include a step where the rat is held by the tail and symmetry of motor behavior is assessed.

**Fig. 1:**
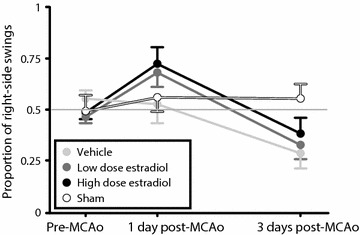
EBST results in a previous study from our lab showing a shift phenomenon. Day 1 post-MCAo, the rats in two out of three stroke groups swung predominantly to the side contralateral to the ischemic hemisphere, but had day 3 post-MCAo developed an ipsilateral side bias. Adapted from Ström et al. [42].

In a previous study from our lab [42], one of the outcomes was the EBST (performed 1 and 3 days after MCAo), and an interesting phenomenon was observed. During testing day 1 post-MCAo, the rats in two out of three stroke groups swung predominantly to the side contralateral to the ischemic hemisphere (from here on, contralateral or ipsilateral refer to direction of swings in relation to the ischemic hemisphere), but had day 3 post-MCAo developed an ipsilateral side bias (Fig. 1). This shift was highly unexpected, and encouraged us to further investigate the phenomenon. Hence, we performed the current study consisting of two steps: first, the literature was systematically searched for studies using the EBST after ischemic stroke in rodents to elucidate if the shift that we observed had been noticed before, and if consensus existed regarding swing direction. Secondly, an experiment was conducted to systematically investigate the suggested shifting behavior in the EBST and further how robust the phenomenon was to alterations in methodology, both regarding sex of the animals and infarct size (occlusion time).

## Methods

### Systematic review

In addition to our abovementioned study (Fig. 1; [42]), the Medline database was searched through PubMed for papers with the EBST using the search string (*stroke or* “*cerebral ischemia*”*) and* (“*elevated body swing*” *or EBST or* “*tail suspension*”). Papers published up until June 2014 were reviewed (n = 36). An article was included if the following criteria were met:

1. Article written in English.
2. Rodents were subjected to unilateral cerebral ischemia.
3. At some point after the induced ischemia, the animals were suspended by their tails and the directions of the head and body movements were recorded.

**Fig. 2:**
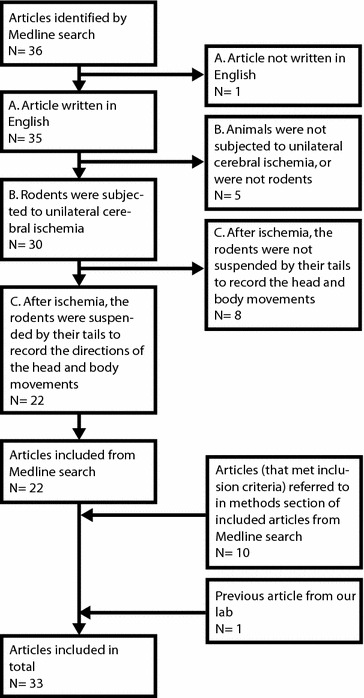
Flow chart of article inclusion for the systematic review. From the original Medline search and through reviewing the articles retrieved, together with the previous article from our lab [42], there were 33 articles. These were further analyzed with regard to main swing direction in the EBST after unilateral cerebral ischemia.

Subsequently, in the included papers retrieved by the Medline search (n = 22), the descriptions of the EBST in the methods sections were searched for references. If criteria were met in these referred articles, they were also included (n = 10). From all the included papers (n = 33; Fig. 2), information regarding whether the stroked animals in the studies swung mainly towards the side contralateral or ipsilateral to the ischemic hemisphere, or if there was no biased swing activity, was extracted. If no statistical comparison had been performed, an explicit statement that the swing activity was biased towards the contralateral or the ipsilateral side was accepted. In cases where no information was provided regarding direction of the biased swing activity, the swing direction was labeled as “unclear”. One group showing biased swing activity was considered enough to label the paper as either contralateral, ipsilateral, unclear or with a shift over time, even though other groups in the same study might have presented without a side preference. Each paper was also scanned for indications of a shift of main swing directions, similar to what was previously observed in our lab (see “Background” and [42]). If a shift was found, the paper was categorized into “shift over time”.

### Animals

Eighty-three adult (age 14 weeks at start of experiment) male and female Sprague–Dawley rats (Taconic Europe, Ry, Denmark) were housed two and two preoperatively (base: 21.5 × 36 cm, height: 18.5 cm) with nesting material (Sizzlenest, Datesand Ltd, Manchester, England) and solitarily postoperatively. The room temperature was 21°C, a 12-h light/dark cycle was maintained and the rats had free access to standard rodent chow (801730, Special Diets Service, Essex, England) and water. All procedures were conducted in accordance with the National Committee for Animal Research in Sweden and Principles of Laboratory Animal Care (NIH publication no. 86-23, revised 1985). The study was approved by the Local Ethics Committee for Animal Care and Use at Linköping University.

### Grouping

**Fig. 3:**
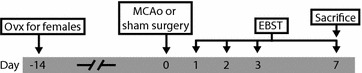
Experimental outline. Fourteen days before MCAo the females were ovariectomized (day −14). On day 0 the animals were subjected to either MCAo or sham operation and on days 1, 2, 3 and 7 the EBST was performed. The animals were sacrificed on day 7.

The rats were randomly allocated into six groups: Four MCAo groups (Male 30 min, n = 13; Male 60 min, n = 20; Male 90 min, n = 18; Female 60 min, n = 13) and two sham groups (Male sham ICA, n = 9; Male sham CCA + ECA, n = 10). See Fig. 3 for an overview of the experiment. Animals of both sexes, as well as different occlusion times were included to enable a broader investigation of EBST behavior.

### Surgical procedures

For ovariectomy, MCAo and sham surgery, the animals were anesthetized with isoflurane (Forene^®^, Abbott Scandinavia AB, Solna, Sweden), 4.5% for induction and 1.5% for maintenance, delivered in a 30/70 mixture of O_2_/N_2_O. Body temperature was maintained at 37°C using a homeothermic blanket connected to a rectal probe (50-7061, Harvard Apparatus, Holliston, MA, USA). Ophtalmic ointment (Lubrithal, VetXX, Uldum, Denmark) was provided for eye protection. For pain relief, 5 mg/kg bodyweight of carpofen (462986, Rimadyl Vet, Pfizer ApS, Ballerup, Denmark) was administered during ovariectomy (only the females were thus subjected to this treatment, but a washout period of 14 days followed before MCAo). Before MCAo and sham surgery, 1.25 mg/kg bodyweight of bupivacaine (Marcain, AstraZeneca, Södertälje, Sweden) was administered. Fluid replenishment with 5 mL saline was given in connection with the MCAo and sham surgery. The skin was cleaned with Iodine solution (Jodopax vet^®^; Pharmaxim AB, Helsingborg, Sweden) before incisions.

14 days prior to MCAo, the female rats (group Female 60 min) were ovariectomized via the dorsal route [43].

The groups Male 30 min, Male 60 min, Male 90 min and Female 60 min were subjected to transient cerebral ischemia in the left hemisphere induced by the filament method, based on the procedure originally described by Koizumi [44] and Longa [45]. With the rat in supine position, a 2 cm cervical midline incision was made and the common (CCA), internal (ICA) and external (ECA) carotid arteries were freed from surrounding tissue. CCA and ECA were ligated with a suture (6-0 silk suture, Johnson & Johnson, New Brunswick, NJ, USA), while ICA was temporarily clipped with a vascular microclip (8 mm artery clip, Rebstock Instruments Gmbh, Dürbheim, Germany). Thereafter, an incision was made in the CCA and a 30 mm silicone coated 4-0 nylon suture (403756, Doccol, Redlands, CA, USA) was inserted and advanced up ICA approximately 18–20 mm until a mild resistance was felt, indicating correct placement. The filament was secured by a knot and the animal was allowed to wake up from anesthesia. After 30 (Male 30 min), 60 (Male 60 min and Female 60 min) or 90 (Male 90 min) minutes of occlusion, during which time the rat was kept in a heated cage (25°C), it was re-anesthetized to enable filament withdrawal, permanent occlusion of ICA and proper closure of the incision. The rats were allowed to recover in heated cages (25°C) for 1 h, and during the first 24 h postoperatively, they were provided water-soaked food pellets in a petri dish on the cage floor to encourage eating. The rats in the sham groups (Male sham ICA and Male sham CCA + ECA) were subjected to the same procedure, with some exceptions. For group Male sham CCA + ECA both CCA and ECA vessels were ligated but no incision was made in CCA and hence no filament was inserted. For group Male sham ICA, only ICA was ligated, no incision was made and no filament was inserted.

### Elevated body swing test

Although EBST is the most common term used to describe the test discussed herein, it has also been called “the tail suspension test” [46]. However, this denomination usually refers to a similar test used for screening antidepressants in mice, in which the degree of immobility is observed rather than the direction of the swings. Yet other names, “C-shaped lateral bending of body” [32] and “hang-tail test” [47], have been used for similar tests.

Before MCAo and on days 1, 2, 3 and 7 (Fig. 3) the EBST was performed for all animals based on the original description by Borlongan et al. [5] with some minor modifications. The rat was put in a transparent cage (36 × 19.5 × 18.5 cm) and allowed to attain a neutral position, with all four paws on the ground. By the base of the tail, it was then elevated to approximately 10 cm from the bottom of the cage and the direction of the first body swing, i.e. a >10° bending of the upper body out of the vertical axis to either side, was recorded. Before the next swing, the animal was placed back in the cage to reposition. Once it was visibly balanced and not displaying a preference for one side, it was re-suspended. The procedure was repeated 20 times. If the animal did not commence swing behavior, the base of the tail was gently pressed in order to induce this. The examiner alternated the hand used to pick up the rat and the position relative to the testing cage to avoid influencing the direction of swings, and the test was carried without any disturbing objects in the immediate surroundings. The proportion of right-side swings were calculated. For baseline values and testing on day 1 the EBST was performed only once per animal, while days 2, 3 and 7 had two testing sessions where the animals were allowed to rest for approximately 30 min in between.

To be able to statistically test the shifting behavior, the following definition was used:

1. Pre-MCAo (baseline), the proportion of right-side swings must not be significantly different from 0.5.
2. On day 1, the proportion of right-side swings should be significantly higher than baseline.
3. On day 2, 3 and 7 the side preference should shift and hence the proportion of right-side swings should be significantly lower than baseline and remain there.

### Infarct measurement

Under anesthesia (see “Surgical procedures” above) the rat was decapitated using a rodent guillotine. The brain was dissected out, cooled in ice water for 5 min and subsequently sliced in 2 mm slices using a rat brain matrix (RBM-4000, ASI Instrument Inc., USA). The slices were then soaked for 15 min in 2% 2,3,5-triphenyltetrazolium hydrochloride solution (TTC; Sigma-Aldrich Sweden AB, CAS# 298-96-4, Stockholm, Sweden) at 37°C and scanned (ScanJet 2c, Hewlett-Packard, Palo Alto, CA, USA). Infarcts were measured in a similar way as described by Goldlust et al. (8784144), with an automatic 40% green spectrum threshold (SigmaScan Pro 5, Systat Software Inc, San Jose, CA, USA). Total infarct volume was calculated indirectly according to Swanson et al. [48]:

Corrected infarct volume = [contralateral hemisphere − [ipsilateral hemisphere − crude infarct]]/contralateral hemisphere.

The indirect method (calculation based on remaining viable tissue in the lesioned hemisphere) was chosen to minimize error introduced by loss of brain tissue due to the infarction 7 days after MCAo. Direct measurement of infarct size (pixels) was used to calculate proportion of infarct in cortex and striatum, respectively.

### Statistics

Based on the definition of the shift (see above), occurrence of shifting behavior in all groups subjected to MCAo was statistically tested by repeated measures ANOVA with post hoc testing against baseline, Bonferroni corrected. The same method was used for analysis after regrouping of males according to infarct size. For group Male small infarcts Greenhouse–Geisser correction was used due to violation of sphericity (tested with Mauchly’s sphericity test). One-sample t tests were used to test if the proportion of right-side swings in the groups at baseline (before MCAo) differed from 0.5.

Comparisons of EBST results (proportion of right-side swings) between the groups Male 60 min and Female 60 min, and between the male groups (Male 30 min, Male 60 min and Male 90 min) were made by two-way mixed ANOVA with day as within-subjects variable and group as between-subjects variable. Because of unequal variances (Levene’s test) in both analyses, data were square root transformed. After square root transformation, Levene’s test was performed again and did not show unequal variances.

One-way independent ANOVA was used to compare infarct volumes in the MCAo groups (Female 60 min, Male 30 min, Male 60 min and Male 90 min). Data was square root transformed because of unequal variances (Levene’s test). After transformation, Levene’s test was performed again and showed no unequal variances. For comparison of the proportion of infarcts in cortex between the MCAo groups (Female 60 min, Male 30 min, Male 60 min and Male 90 min), the non-parametric equivalent to one-way independent ANOVA Kruskal–Wallis test was used since variances were unequal (Levene’s test) both before and after square root transformation. Homogeneity of variance for the Kruskal–Wallis test was confirmed by one-way independent ANOVA for the absolute difference between ranks and mean ranks of the groups.

To compare the shifting animals with the non-shifting regarding infarct volume and location of infarct (proportion of infarct in cortex), two two-way ANCOVA models were performed. To control for sex and occlusion time, these variables were included as a fixed categorical factor and a continuous covariate, respectively.

Data are presented as mean ± SEM throughout the article.

### Protocol violations

The overall mortality of the study was 29% (24/83, Table 1). All animals that died spontaneously prior to sacrifice on day 7 were excluded from the analyses.

**Table 1:** Mortality in experiment

Group	Mortality day −14 to 7
Male 30 min	3/13 (23%)
Male 60 min	10/20 (50%)
Male 90 min	8/18 (44%)
Female 60 min	3/13 (23%)
Male sham ICA	0/9 (0%)
Male sham CCA + ECA	0/10 (0%)
Total	24/83 (29%)

Animals that did not have any visible infarcts with direct measurement (for calculation of proportion of infarct in cortex and striatum, respectively) but still had a positive value for total infarct volume which was calculated with another approach (indirectly according to Swanson [48], described above) were excluded in the analyses of cortex/striatum ratio and differences in proportion of infarcts in cortex. With indirect infarct measurement it is possible to obtain negative values and, as mentioned above, this method was used for calculation of total infarct volume. In 13 animals with very small infarcts, negative values were set to 0 in the analysis.

Actual group sizes after exclusion of animals that died was n = 10 for all the MCAo groups, and n = 9 and n = 10 for the Male sham ICA and Male sham CCA + ECA groups, respectively. For analyses of cortex/striatum ratio and differences in proportion of infarcts in cortex, however, group sizes were Female 60 min (n = 9), Male 30 min (n = 10), Male 60 min (n = 10), Male 90 min (n = 9), Male sham ICA (n = 8) and Male sham CCA + ECA (n = 5) since some animals were excluded as described in the preceding paragraph.

## Results

### Systematic review

Although swing activity to the side contralateral to the ischemic hemisphere dominated, a few articles reported ipsilateral swings (14/33 and 3/33 respectively, Table 2). Almost half of the studies (14/33, Table 2) were labeled unclear, i.e. a biased swing activity was described but not whether it was ipsilateral or contralateral. The use of females exclusively was reported in only two of the included papers, hence no comparisons regarding sex differences could be performed. One study, except from our previous, reported a shift in direction over time (Table 2). All studies had at least one group with biased swing activity.

**Table 2:** Number of previous articles reporting different directions of biased swing activity [reference in square brackets]

Ipsilateral	Contralateral	Unclear	Shift over time
Male			
2 [8, 21]	9 [9, 11, 15, 16, 18, 20, 22, 30, 34]	13 [10, 12–14, 17, 19, 23, 25–27, 29, 31, 35]	1 [36]
Female			
0	1 [24]	0	1 [42]
Unspecified sex or mixed sexes			
1 [7]	4 [33, 37–39]	1 [28]	0
Total			
3 [7, 8, 21]	14 [9, 11, 15, 16, 18, 20, 22, 24, 30, 33, 34, 37–39]	14 [10, 12–14, 17, 19, 23, 25–29, 31, 35]	2 [36, 42]

### Elevated body swing test

Of all the groups, only Sham ICA had baseline values for proportion of right-side swings that differed significantly from 0.5 [t(8) = −2,69; p = 0.028].

**Fig. 4:**
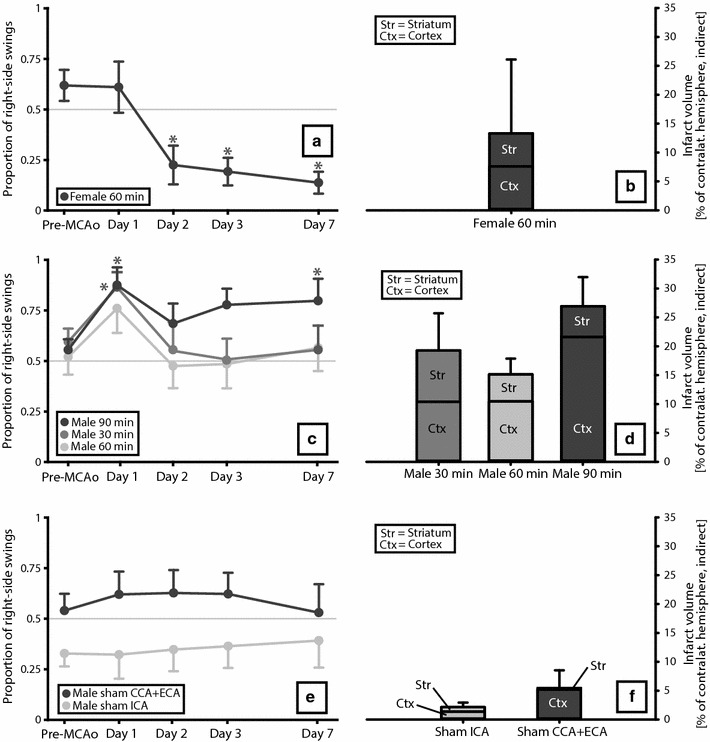
EBST performance (**a**,** c**,** e**) and infarct volumes including location of infarcts (**b**,** d**,** f**). There was a significant difference between the EBST curve for group Female 60 min (**a**) and Male 60 min (**c**) but not for the three male groups (Male 30 min, Male 60 min and Male 90 min). None of the MCAo groups (Male 30 min, Male 60 min, Male 90 min and Female 60 min) differed significantly in infarct volume of location of infarct (proportion of infarct in cortex). *Asterisks* indicate significant difference in proportion of right-side swings compared to baseline values of the group (p < 0.05). Mean ± SEM.

When occurrence of shifting behavior was tested according to the definition (see “Methods”), a main effect of time on proportion of right-side swings was found in the groups Female 60 min [F(4, 28) = 8.63; p < 0.0005], Male 30 min [F(4, 36) = 4.01; p = 0.009] and Male 90 min [F(4, 32) = 5.74; p = 0.001], but not in groups Male 60 min [F(4, 36) = 1.69; p > 0.05], sham ICA [F(4, 32) = 0.22; p > 0.05] and sham CCA + ECA [F(4, 32) = 0.93; p > 0.05]. Post-hoc testing against baseline revealed for group Female 60 significantly lower values on days 2 (p = 0.008), 3 (p = 0.04) and 7 (p = 0.04) (Fig. 4a). Group Male 30 min had increased proportion of right-side swings compared to baseline on day 1 (p = 0.004), and group Male 90 min on day 1 (p = 0.02) and day 7 (p = 0.008) (Fig. 4c).

**Fig. 5:**
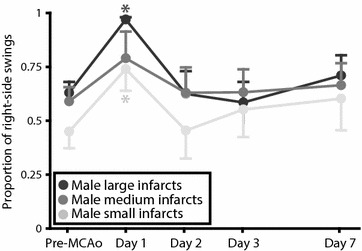
EBST performance in males regrouped according to infarct size. Because of high variability in infarct sizes, the males were regrouped according to infarct size (Male small infarcts, Male medium infarcts and Male large infarcts) in an additional analysis to reflect actual severity of lesions produced rather than occlusion times. *Asterisks* indicate significant difference in proportion of right-side swings compared to baseline values of the group (p < 0.05).

Because of high variability in infarct sizes, an additional analysis of the male rats were done after regrouping according to infarct size (Male small infarcts, Male medium infarcts and Male large infarcts; n = 10; Fig. 5). These groups were, similar to what was described for the original groups above, tested regarding proportion of right-side swings according the definition of shifting behavior. A main effect of time was found for groups Male small infarcts [F(2, 16) = 3,70; p = 0.048] and Male large infarcts [F(4, 36) = 4,92; p = 0.003] but not for group Male medium infarcts [F(4, 36) = 0.73; p > 0.05]. Post-hoc testing against baseline showed significantly higher proportion of right-side swings on day 1 for groups Male small infarcts (p = 0.008) and Male large infarcts (p = 0.004).

The female 60 min group differed significantly from the Male 60 min group regarding proportion of right-side swings over time [F(1, 18) = 6.17; p = 0.023] while no significances were found when the three male groups were compared in the same way [F(2, 27) = 1.62; p > 0.05].

**Fig. 6:**
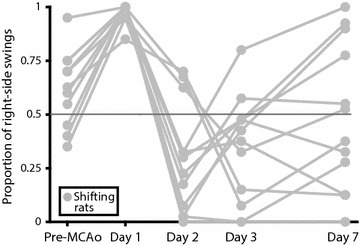
EBST performance of rats displaying a shift. EBST performance of rats from MCAo groups (Male 30 min, Male 60 min, Male 90 min and Female 60 min) displaying a shift in direction (proportion of right-side swings >0.8 on day 1 and <0.4 on days 2 or 3).

Even though no significant shifting behavior was seen on a group level, a separate graph was constructed to illustrate that the phenomenon existed among several individuals. From MCAo groups (Male 30 min, Male 60 min, Male 90 min and Female 60 min), individual rats with proportion of right-side swings >0.8 on day 1 and <0.4 on days 2 or 3 were plotted (Fig. 6). With this approach, 12/40 rats (30%) of the rats met the criteria (3 from Male 30 min, 5 from male 60 min, one from Male 90 min and 3 from Female 60 min). As comparison, when the criteria were inverted (proportion of right-side swings <0.2 on day 1 and >0.6 on days 2 or 3), only one rat was included.

### Infarct volume

No significant differences in infarct volume [F(3, 36) = 1.46; p > 0.05] or location of infarct (proportion of infarct in cortex; Kruskal–Wallis test; p > 0.541) were seen between the groups that underwent MCAo.

In the analysis of the shifting animals in Fig. 6 versus the non-shifting animals, no significant effect of shift was seen on infarct volume [F(1, 35) = 0.01; p > 0.05] or location of infarct [proportion of infarct in cortex; F(1, 31) = 0.92; p > 0.05].

## Discussion

The systematic review revealed that different articles reported diametrically different swing directions in the EBST after unilateral cerebral ischemia, emphasizing the confusion that exists regarding this test. When the shifting behavior, suggested in the preceding study from our lab [42], was statistically tested according the definition presented in “Methods”, no significant change in direction was seen in any of the groups. However, the side preference profile of the group designed based on the previous study [42] (Female 60 min) still is interesting; a persistent ipsilateral side preference developed but not until day 2. Since rats subjected to MCAo usually are most affected early after stroke, the biggest impact on behavior would have been expected on day 1. Figure 6, with EBST data from shifting animals plotted (data selected post-experiment in such a manner must obviously be interpreted with great caution), shows that although not strong enough to have any major influence on the group curves, a clear shift in swing direction existed among almost a third of the rats. However, this shift was not persistent; on day 7 the proportion of right-side swings varies greatly among the rats. One article in the systematic review also reported a change in direction. After right-sided cerebral ischemia in gerbils, the animals displayed right-biased swings (ipsilateral) on the day of ischemia that switched to left-biased swings (contralateral) at 2 days post-ischemia [36]. Thus, the shift had a similar temporal pattern to the one we described but strangely enough it went in the opposite direction, from ipsilateral to contralateral rather than the other way around. Another study from the systematic review did mention the fact that direction of swings when performing the EBST can differ, and location of ischemia was here proposed as the explanatory factor; striatal damage would result in ipsilateral swings whereas cortical or combined damage would make the animals swing contralaterally [14]. Based on this, the group Female 60 min (that displayed partial ipsilateral swing tendency) in the current study would have been expected to have a smaller proportion of infarct in cortex. However, we did not find any significant differences between the groups regarding infarct location (proportion of infarct in cortex) or infarct volume. Nor were there any significant differences in the explorative comparison of animals with a shift (Fig. 6) versus non-shifting animals for these two outcomes, further challenging location of infarct as the crucial factor determining swing direction. Because of large variability in infarct sizes in the present study, the male MCAo rats were regrouped according to lesion volume in an additional analysis (Fig. 5). It could be argued that such an approach is more relevant than occlusion time since this reflects the actual sizes of the lesions produced rather than the intentions. No shifting behavior according to the definition was found in these groups either, and no obvious pattern regarding infarct size and swing direction was found. Both the group with the smallest infarcts (Male small infarcts) and the group with the largest infarcts (Male large infarcts) had significantly higher proportion of right-side swings compared to baseline on day 1, while the rats with middle-sized infarcts (Male medium infarcts) had no significant differences compared to baseline on any of the post-MCAo days. TTC staining 1 week after the MCAo was used in the current study. Although this has been done before [49, 50], concerns have been raised regarding the use this method at such a late point in time [51].

ECA supplies tissues around the face of the rat and ligation can cause ischemia in this region [52]. Since two different sham groups were used in the current study, one in which we ligated CCA and ECA during the ischemia induction procedure and one with only ICA ligated, we were able to investigate whether the facial ischemia, by affecting EBST behavior, could have anything to do with the observed shift. For example it could have been that rats with facial ischemia were more prone to swing in the opposite direction (contralateral) early after stroke and that this effect decreased over time resulting in a change in preferred swing direction (neutral or ipsilateral). However, although the curves of the two sham groups in Fig. 4e are somewhat different, the proportion of right-side swings did not change distinctly before versus after surgery in either group and therefore the swing phenomenon seen in the MCAo rats is unlikely to be explained by a mechanism related to ECA ligation and facial ischemia.

In the clinic, a process somewhat similar to a shift can sometimes occur after a stroke. Initially, patients typically suffer from flaccid paralysis which in some patients can develop into spasticity after a few weeks [53]. However, this mechanism is an unlikely explanation for several reasons. First, our shift occurs over days rather than weeks. Secondly, spasticity primarily affects limbs [53], while the current phenomenon seems related to axial muscles. And finally, a transition from flaccid paralysis to spasticity should in theory produce a change from ipsilateral to contralateral swings, i.e. the opposite to what we observed, since ischemia affects the part of the body contralateral to the infarcted hemisphere.

To summarize the discussion regarding possible mechanisms behind a shift, three suggestions were presented: location of infarct, ischemia in the ECA territory and spasticity. No support was found in this study for the former two, and the latter seems theoretically unlikely.

The most striking result of the experimental part is that the EBST curve for the female rats is noticeably different from the male groups, even when sex is the only differentiating factor (groups Female 60 min versus Male 60 min; Fig. 4). Also, when compared against group baseline values for proportion of right-side swings, the female group (Female 60 min) presented with significantly lower values on all timepoints except the first, while the two of the male groups, Male 30 min and Male 90 min, on the contrary had values significantly higher than baseline on one and two timepoints, respectively. The fact that we earlier obtained similar results with females [42], corroborates the notion of an effect of sex on EBST performance. The fact that the females are ovariectomized makes estrogens less likely as an explanation for this. Differences in other hormones such as testosterone, or in brain wiring between female and male rats could perhaps contribute, but these suggestions are merely speculations. Unfortunately, only two studies [24, 42] from the systematic review reported the use of females exclusively and therefore it is hard to draw any conclusions from this. However, it is worth mentioning that one of these two studies, the one not performed our lab, had contralateral swings [24], contradicting the abovementioned idea of a sex effect.

### Strengths and weaknesses

A drawback of this study is the mortality, which was higher than what we have seen previously in our lab. Perhaps this was a consequence of intense EBST testing; even though the procedure is not long in duration it is probably stressful for the animal to repeatedly be suspended upside-down. To try to reduce the stress imposed on the rats early after stroke, only one testing session was performed on day 1, whereas the rats were tested twice on days 2, 3 and 7. Cerebral blood flow measurement to ensure successful filament insertion and occlusion was not used which might have decreased the success rate of the ischemia inductions.

A strength is that we included several groups that differed in terms of sex and occlusion time. This made it possible to show how the EBST performance can change with altered conditions. The inclusion of a thorough systematic review of the existing literature on the subject also adds weight to the study.

## Conclusions

The present study shows, both experimentally and through systematic literature review, that the direction of biased swing activity in the EBST for rodents after cerebral ischemia can differ and even shift over time in some situations. Although it does not provide an explanation to why this is the case, it highlights the importance of clear and adequate reporting of behavioral tests for lateralization. Since we could not find a swing profile consistent among all groups, not even if only the first day post-MCAo (day 1) was considered, we cannot recommend the use of this test for motor asymmetry after MCAo in rats.

## Abbreviations

EBST: elevated body swing test
MCAo: middle cerebral artery occlusion
CCA: common carotid artery
ICA: internal carotid artery
ECA: external carotid artery
TTC: 2,3,5-triphenyltetrazolium hydrochloride
